# Revitalizing Pediatric Resident Educational Conferences Using Kotter’s Eight-Step Change Model

**DOI:** 10.7759/cureus.117744

**Published:** 2026-10-10

**Authors:** Elizabeth L Nguyen, Jennifer Sedler, Ryan R Lion, Victor Ritter, Caroline Rassbach

**Affiliations:** 1 Department of Pediatric Hospital Medicine, David Geffen School of Medicine at University of California, Los Angeles (UCLA), Los Angeles, USA; 2 Department of Pediatric Hospital Medicine, University of Colorado School of Medicine, Aurora, USA; 3 Department of Pediatric Oncology, St. Jude Children’s Research Hospital, Memphis, USA; 4 Department of Medicine, Quantitative Sciences Unit, Stanford University School of Medicine, Palo Alto, USA; 5 Department of Pediatrics, Lucile Packard Children's Hospital Stanford, Stanford University School of Medicine, Palo Alto, USA

**Keywords:** change management, medical education, morning report, noon conference, resident education

## Abstract

Objective: Resident educational conferences (i.e., morning report and noon conference) serve to increase clinical knowledge and to shape educational culture within a program. During the COVID-19 pandemic, dwindling conference attendance, decreasing participation, and a perception that formal educational time was less protected were observed at our institution, highlighting a need for culture change around trainees’ educational time. Our objective was to apply Kotter's eight-step Change Model (KCM) to increase attendance and improve residents' perception of the culture of educational conferences in their residency program.

Methods: This was a mixed-methods study involving pediatric residents at a large, university-affiliated children’s hospital in Palo Alto, California, United States from 2022 to 2023. A total of 113 residents were eligible to participate. We communicated the need and vision for change with stakeholders, recruited conference champions, and addressed resident-identified barriers to help facilitate attendance. Attendance was recorded daily, and residents were surveyed bi-monthly, evaluating their perception of culture using Likert scales and free-response questions. A Wilcoxon signed-rank test was used to compare pre- and post-intervention weekly median conference attendance, and change in the Conference Culture Survey was assessed using ANOVA F-tests.

Results: Weekly median conference attendance increased by 107% from 15 to 31 (p<0.001). Pre-intervention survey response rate was 41% (n=46), while post-intervention survey response rates were 42-67% (range, 47-76). Residents perceived meaningful improvements in conference culture (p<0.001), protection from clinical obligations (p<0.001), and board exam preparation (p<0.001).

Conclusion: Over a 12-month period, attendance and perception of educational conference culture improved. KCM is a useful tool that can guide educators in intentionally addressing conference educational culture within their own contexts.

## Introduction

During the COVID-19 pandemic, pediatric trainee education faced significant challenges [1-3]. Patient encounters decreased, and the shift to virtual didactics disrupted shared norms around where, how, and with what level of engagement residents participated in educational conferences. During this same period, declining first-time pass rates for the American Board of Pediatrics Initial Certifying Examination raised concern about the broader impacts of these disruptions on trainee education [4].

Protected educational time in the form of morning report and noon conferences is a time-honored tradition residents have ranked as a critical educational activity during training [5]. These conferences serve not only to increase clinical knowledge, but also to provide shared, scheduled spaces where expectations around participation, engagement, and prioritization of education are enacted and reinforced [6,7]. In this paper, we use the term “conference culture” to describe the shared norms and expectations around learning in the setting of morning report and noon conferences, including attendance, engagement, and protection from competing clinical demands. Pandemic-related disruptions to educational conferences may have affected these shared norms and expectations, with implications that extend beyond conference attendance to the learning culture conferences are meant to support.

Prior efforts to improve conference attendance and participation have largely been fragmented, often targeting single barriers residents face rather than the broader educational culture in which conferences are embedded. For example, some studies have addressed conference content, e.g., focusing on high-yield clinical topics, standardizing components of morning report, encouraging an interactive session, and implementing a formal assessment tool, to improve the educational value of the conference [8-10]. Other approaches, including offering complimentary food to incentivize attendance, have led to modest yet statistically significant increases in attendance [11]. What remains underexplored is how a structured, theory-informed change management approach can be applied to deliberately reshape and improve norms around attendance and engagement within residency conferences.

Kotter’s eight-step Change Model (KCM) is a framework for leading organizational change and consists of eight iterative stages: (i) establish a sense of urgency, (ii) create a guiding coalition, (iii) develop a vision and strategy, (iv) communicate the change vision, (v) empower broad-based action, (vi) generate short-term wins, (vii) consolidate change and produce more wins, and (viii) anchor new approaches in the culture [12]. Originally developed and used mainly outside healthcare, KCM has more recently been adopted within healthcare settings to improve patient care and safety [13-16]. While KCM has grown in popularity as a method taught to lead change, few studies have formally used KCM to create a change in medical education [17-20]. Specifically, some medical education programs have applied KCM to curricular reform, demonstrating improved learner satisfaction, engagement, and perceived preparedness for board examinations [21,22]. Given its emphasis on stakeholder alignment and norm-setting, KCM offers a potentially useful framework for addressing the complex, culture-level challenges facing post-pandemic residency education.

During the 2021 Annual Program Evaluation of the pediatric residency program at Stanford University, the Accreditation Council for Graduate Medical Education (ACGME) Resident Survey identified two areas for improvement: achieving an appropriate balance between education (e.g., clinical teaching, conferences, lectures) and patient care and establishing protected time to participate in structured learning activities [23,24]. At Stanford University, the transition to virtual conferences during the pandemic allowed pediatric residents to attend from clinical workrooms where they remained accessible for patient care responsibilities and were frequently interrupted by competing clinical demands. Over time, clinical teams became accustomed to residents remaining available during scheduled conference time, while incoming resident classes had limited exposure to the previous in-person conference routines. Despite repeated efforts by residency leadership to encourage conference participation, low attendance persisted in the months preceding our initiative. These observations were accompanied by declining average In-Training-Exam (ITE) scores and certification exam pass rates. Together, these findings highlighted concerns spanning accreditation signals, learner engagement, conference culture, and educational outcomes, and reinforced an urgent need to strengthen the educational environment. In the pediatric residency program at Stanford University, resident conferences offered a feasible and visible setting in which these interrelated concerns could be examined and addressed.

In response to the pandemic-related disruptions to educational culture at our institution, we applied a structured change management framework to our resident didactics. The purpose of this study was to examine whether applying KCM was associated with changes in conference attendance and residents’ perception of conference culture.

## Materials and methods

Setting and participants

This was a mixed-methods study that included all 113 residents at Stanford University's pediatric residency program from January 2022 to May 2023. Residents in this program rotate across multiple clinical sites, including a large quaternary-care, university-affiliated, free-standing children’s hospital where morning report and noon conference physically take place, a pediatric unit within a county hospital, and several outpatient clinical settings. As a result, attendance at conferences reflects both cultural factors, such as shared expectations surrounding conference attendance and protection of educational time, and structural factors, such as clinical schedules and rotation sites that influence residents’ ability to attend scheduled educational conferences. The study was designed and implemented by the program’s chief residents as part of their leadership role. The Stanford University School of Medicine’s Institutional Review Board (IRB) determined that the study did not meet the definition of human subject research as defined in federal regulations 45 CFR 46.102 or 21 CFR 50.3 and that no further IRB review was required (protocol number: 66956).

Interventions

Our approach to changing resident educational conference culture was informed by KCM [12], as illustrated in Figure 1. We used the framework to guide our change process. As a result of following KCM steps, we identified barriers and facilitators that were able to be addressed by specific interventions. These interventions were implemented and supported by the KCM process.

**Figure 1:**
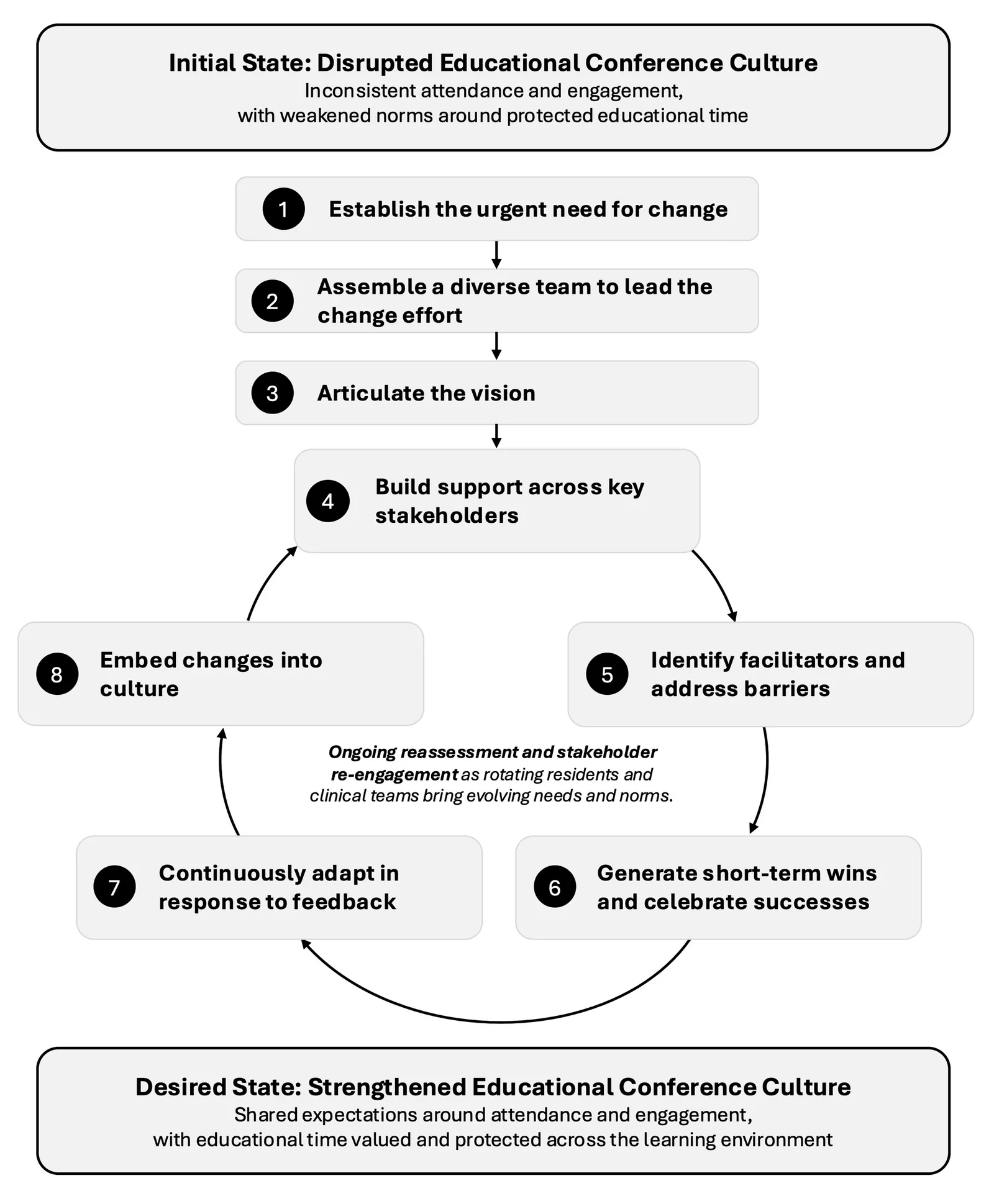
KCM-informed approach to changing resident educational conference culture KCM: Kotter’s 8-Step Change Model [12] The figure was created by the authors using PowerPoint (Microsoft Corporation, Redmond, Washington, United States)

Step 1

We established an urgent need for change by utilizing data regarding decreasing board exam pass rates, decreasing ITE scores, low conference attendance, and low conference satisfaction to demonstrate the need for change.

Step 2

We assembled a diverse team to lead the change effort that included the residency program director, an associate program director, residency coordinators, a statistician, a resident liaison, and attending physicians in addition to the chief residents.

Step 3

We articulated our strategic vision to remove barriers and implement facilitating factors to improve conference attendance and engagement, resident perception of conference culture, and resident clinical reasoning skills.

Step 4

To build support for the vision across key stakeholders, we met with leadership across the residency, department, and institution. This included discussions with the Department Chair of Pediatrics and the Executive Leadership team to establish institutional buy-in; Division Chiefs and residency rotation directors to encourage faculty to support residents’ conference attendance; and the Senior Vice President of Strategic Space Planning and nursing leadership to address barriers related to conference space and protected educational time. We also presented our vision to the residents to ensure they understood our goals.

Step 5

To identify facilitators and remove barriers influencing conference attendance, we administered a pre-intervention Conference Culture Survey (“survey”) and conducted a force field analysis (Figure 2). Based on the identified barriers and facilitators, we co-developed targeted interventions with the guiding coalition and partnered with relevant stakeholders to implement them.

**Figure 2:**
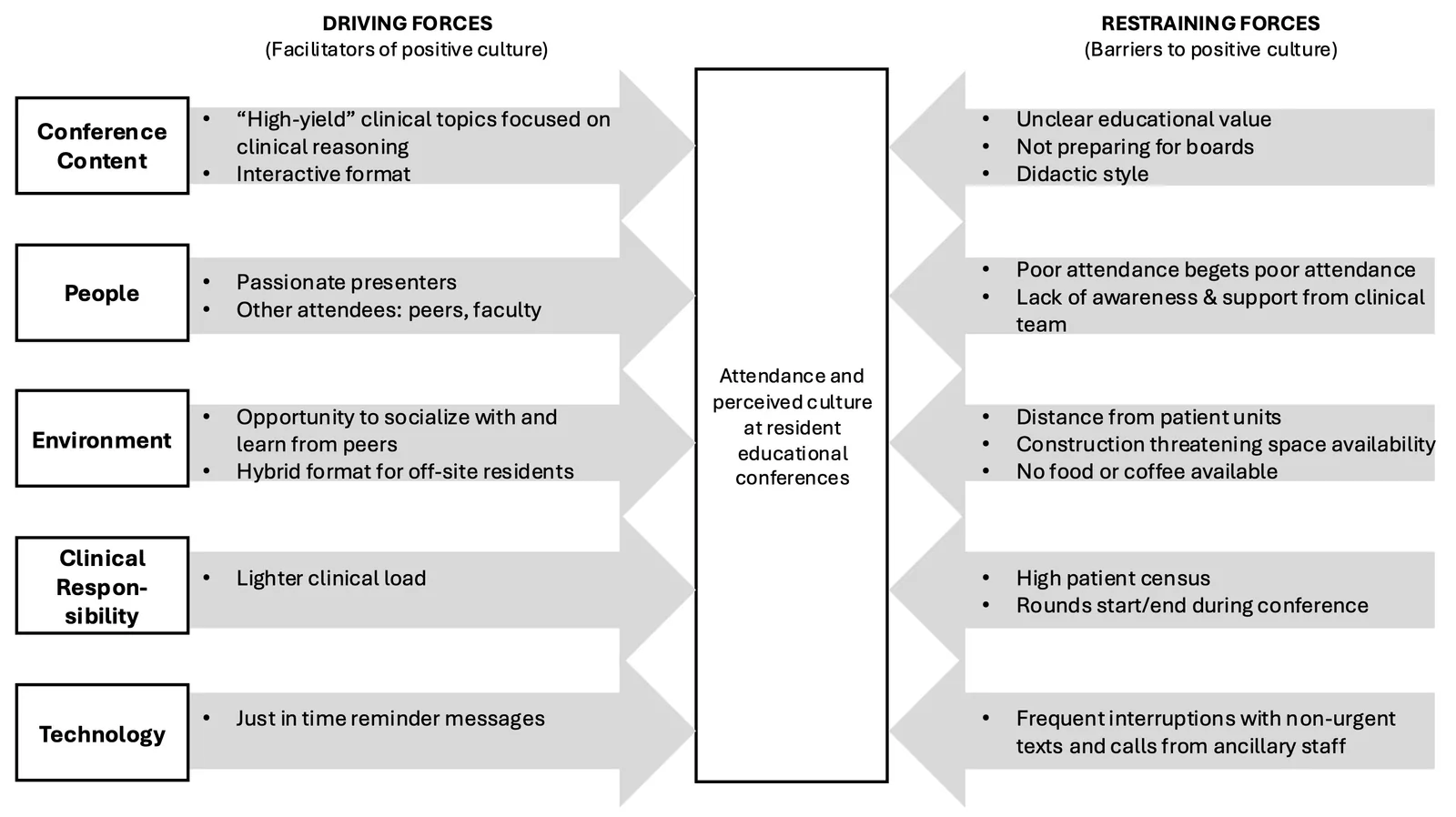
Force field analysis of driving and restraining forces contributing to decreased resident educational conference attendance and poor perception of conference culture The figure was created by the authors using PowerPoint (Microsoft Corporation, Redmond, Washington, United States)

Step 6

We followed up the results of those interventions with subsequent surveys to evaluate short-term wins, which we shared with residents and faculty to celebrate successes and sustain engagement.

Step 7

Approximately every two months, we reassessed outcomes and adapted our interventions based on feedback.

Step 8

By regularly assessing change at this interval and through this iterative process, we sought to reinforce this initiative and embed attendance and participation into our residency culture to promote sustainability beyond the one-year term of chief residents.

Highlights of interventions included improving conference content, cultivating support from residents’ clinical teams, and enhancing the conference environment. A complete list of interventions performed can be found in Appendix A. 

Measures

The primary outcomes assessed included attendance trends and perceived conference culture. We recorded attendance at conferences daily. Pre-intervention data (January 2022-May 2022) were obtained retroactively from Zoom (Zoom Communications, Inc., San Jose, California, United States) for conferences held virtually at that time. In the post-intervention period (June 2022-May 2023), we used Zoom to pull virtual attendance and collected in-person attendance by badge scanning or manual entry into a Microsoft Excel spreadsheet (Microsoft Corporation, Redmond, Washington, United States).

To understand resident perception of conference culture over time, we conducted five surveys over the year using REDCap electronic data capture tools hosted at Stanford University [25,26]. The first survey was e-mailed to the residents pre-intervention, and the subsequent four were administered every two months. Each survey featured the same 25 prompts on the perceived value of educational conferences, factors influencing attendance, feedback on recent interventions, and suggestions for improvement (see Appendix B). We measured responses on a five-point Likert scale, where 1 represented “strongly disagree” and 5 represented “strongly agree.” The study team developed the survey iteratively, generating questions based on the study objectives. To establish content and response process validity, we reviewed the survey with medical education and survey methodology experts and refined it through pilot-testing and cognitive interviewing with three faculty and two external residents.

Lastly, we compared the program's annual ACGME surveys before and after the intervention to assess perceptions about protected time for structured learning activities.

Analysis

Because residents rotate through different clinical services and sites on a weekly basis, the weekly median attendance was calculated to account for expected variation in the number and composition of residents available to attend conferences. A Wilcoxon signed-rank test was used to compare median attendance between the pre-intervention and post-intervention periods. To assess changes in perceptions of conference culture, responses from the five surveys were analyzed, mean scores were calculated for each item, and differences across time points were assessed using ANOVA F-tests. To account for multiple comparisons across surveys, a Bonferroni correction was applied to adjust the threshold of significance and reduce the risk of type I error. Demographic characteristics were compared across survey time points using Fisher’s exact test for gender identity and Pearson’s chi-square test for level of residency training. Because respondents could select more than one race/ethnicity, each race/ethnicity category was evaluated separately across survey time points using Fisher’s exact test. All statistical analyses were performed using R version 4.4.3 (R Foundation for Statistical Computing, Vienna, Austria).

## Results

Attendance

The total number of residents included in the study was 113. The median number of conference attendees during the pre-intervention period was 15. Following the intervention, the weekly median attendance increased to 31 attendees (p<0.001). A visualization of attendance trends over time is provided in Figure 3. Attendance fluctuated over the course of the intervention and was highest at the beginning of the academic year. However, despite these fluctuations, attendance after interventions remained significantly higher compared with the pre-intervention period.

**Figure 3:**
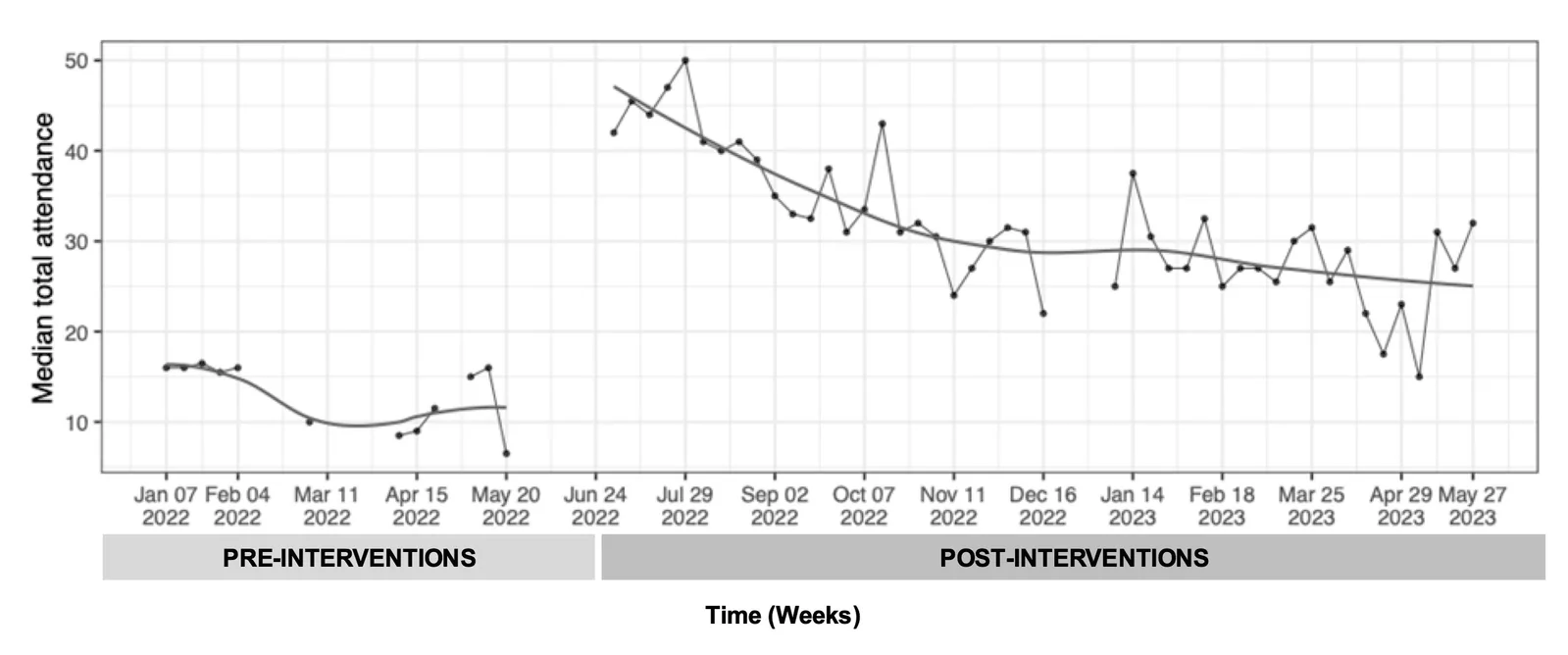
Weekly Median Conference Attendance January 2022 - May 2023 The study population included 113 residents. The number of residents expected to attend conferences varied daily based on clinical schedules, rotation site, and competing educational activities.

Conference culture survey

Survey response rate varied over time, with 41% (n=46) pre-intervention and between 42% (n=47) and 67% (n=76) in surveys post-intervention. Demographic characteristics were similar across the five survey time points (Table 1). Fisher’s exact tests demonstrated no significant differences in gender identity or individual race/ethnicity categories across time points, while Pearson’s chi-square test demonstrated no significant difference in level of residency training.

**Table 1:** Demographics of respondents to each conference culture survey Participants were able to select all applicable race/ethnicities, so the percentage breakdown will not sum to 100%. PGY: post-graduate year

Demographic Characteristics		Pre-Intervention (June 2022), (n=46)		Post Intervention (Sept 2022), (n=76)		Post Intervention (Nov 2022), (n=48)		Post Intervention (Jan 2023), (n=54)		Post Intervention (May 2023), (n=47)	
		Frequency	Percentage	Frequency	Percentage	Frequency	Percentage	Frequency	Percentage	Frequency	Percentage
Gender Identity	Female	32	69.6	56	73.7	38	79.2	42	77.8	37	78.7
	Male	13	28.3	17	22.4	8	16.7	11	20.4	9	19.1
	Gender Non-conforming	0	0.0	0	0.0	0	0.0	0	0.0	0	0.0
	Gender Non-binary	0	0.0	0	0.0	0	0.0	0	0.0	0	0.0
	Prefer not to answer	1	2.2	3	3.9	2	4.2	1	1.9	1	2.1
Level of Residency Training	PGY-1	15	32.6	29	38.2	14	29.2	19	35.2	15	31.9
	PGY-2	17	37.0	26	34.2	18	37.5	19	35.2	16	34.0
	PGY-3 or higher	14	30.4	21	27.6	16	33.3	16	29.6	16	34.0
Race/Ethnicity	American Indian or Alaska Native	0	0.0	2	2.6	2	4.2	0	0.0	2	4.3
	Asian	13	28.3	25	32.9	12	25.0	20	37.0	13	27.7
	Black or African American	3	6.5	2	2.6	1	2.1	2	3.7	1	2.1
	Hispanic or Latino	5	10.9	11	14.5	9	18.8	7	13.0	4	8.5
	Native Hawaiian or Pacific Islander	1	2.2	1	1.3	0	0.0	1	1.9	1	2.1
	White	19	41.3	34	44.7	22	45.8	24	44.4	22	46.8
	Other	2	4.3	0	0.0	0	0.0	1	1.9	1	2.1
	Prefer not to answer	5	10.9	9	11.8	6	12.5	4	7.4	6	12.8

Table 2 presents the mean responses to survey questions over time. There were significant improvements in mean scores for items 1-9, which assessed perspectives on the conference experience, with most items showing progressive improvements over time. Items 10-14, which pertain to factors influencing attendance, showed no significant differences over time.

**Table 2:** Mean resident responses to each survey item across survey time points Responses were measured using a 5-point Likert scale (1= “strongly disagree”, 5 = "strongly agree”), where lower means indicate stronger disagreement with the statements regarding the culture of morning report and noon conference. We analyzed differences in perceptions of conference culture across time points using ANOVA F-tests. To account for multiple comparisons across surveys, we applied a Bonferroni correction to adjust the threshold of significance and reduce the risk of type I error.

Survey Item	Pre-Intervention (June 2022) (n=46), mean±SD	Post-Intervention (September 2022) (n=76), mean±SD	Post-Intervention (November 2022) (n=48), mean±SD	Post-Intervention (January 2023) (n=54), mean±SD	Post-Intervention (May 2023) (n=47), mean±SD	ANOVA F-statistic	Bonferroni P-Value
1. Morning report/noon conference is a valuable use of my time.	3.48±1.17	4.13±0.82	4.31±0.75	4.28±0.74	4.32±0.69	8.4	<0.001
2. We have a good culture of morning report/noon conference attendance.	1.70±0.66	3.70±0.89	3.50±0.95	3.69±0.89	3.77±0.98	48.07	<0.001
3. I am motivated to try to attend morning report/noon conference.	3.00±1.23	3.92±0.99	3.88±0.96	4.17±0.91	4.11±0.76	10.99	<0.001
4. I enjoy attending morning report/noon conference.	3.63±1.00	4.14±0.78	4.25±0.79	4.41±0.77	4.38±0.68	7.35	<0.001
5. Our morning report/noon conference time feels protected from clinical obligations.	1.65±0.82	2.95±1.01	2.90±1.06	2.81±1.07	3.06±0.94	16.28	<0.001
6. Our morning report/noon conferences are educational.	3.83±0.80	4.33±0.72	4.40±0.61	4.43±0.60	4.55±0.50	8.42	<0.001
7. Our morning report/noon conferences are interactive.	3.50±0.72	4.09±0.75	4.27±0.64	4.17±0.69	4.32±0.52	10.98	<0.001
8. Our morning report/noon conferences are fun.	3.47±0.76	4.00±0.89	4.15±0.77	4.19±0.73	4.19±0.58	7.42	<0.001
9. Our morning reports/noon conferences are preparing me for my pediatric board exams.	2.85±1.07	3.63±0.85	3.77±0.72	3.93±0.75	4.06±0.70	15.47	<0.001
10. I am more likely to attend an in-person morning report/noon conference.	3.46±1.00	3.93±1.05	4.08±1.01	4.00±1.05	4.00±0.98	2.81	0.361
11. I am more likely to attend a Zoom/virtual morning report/noon conference.	2.91±0.89	2.78±1.14	2.69±1.01	2.89±1.11	2.85±1.16	0.36	1.00
12. I am more likely to attend a morning report/noon conference if it’s a topic I’m interested in.	4.15±0.73	4.24±0.80	4.33±0.66	4.17±0.82	4.26±0.77	0.45	1.00
13. I am more likely to attend a morning report/noon conference if I know faculty/attendings are attending.	3.39±1.11	3.41±0.98	3.17±1.08	3.19±0.95	3.45±0.93	0.91	1.00
14. I am more likely to attend morning report/noon conference if there is coffee.	4.11±0.97	3.89±1.10	3.85±1.11	3.70±1.19	3.79±1.21	0.89	1.00

While multiple survey items showed significant changes, the three with the most pronounced improvements were: Item 2 (“We have a good culture of morning report and noon conference attendance”), Item 5 (“Our conference time feels protected from clinical obligations”), and Item 9 (“Our conferences are preparing me for the pediatrics board exams”) (Figure 4). Perceptions of positive conference culture (Item 2) improved significantly (F=48.07, p<0.001), with the mean rising from 1.7 (89% disagreeing) pre-intervention to 3.5-3.77 post-intervention; by year’s end, perceptions flipped, and 72% agreed or strongly agreed. Protected time (Item 5) also improved (F=16.28, p<0.001); disagreement dropped from 83% to 21%, while agreement rose from 2% to 34%. Board preparation (Item 9) showed similar gains (F=15.47, p<0.001), shifting from 39% disagreement and 37% agreement at baseline to just 2% disagreement and 83% agreement post-intervention.

**Figure 4:**
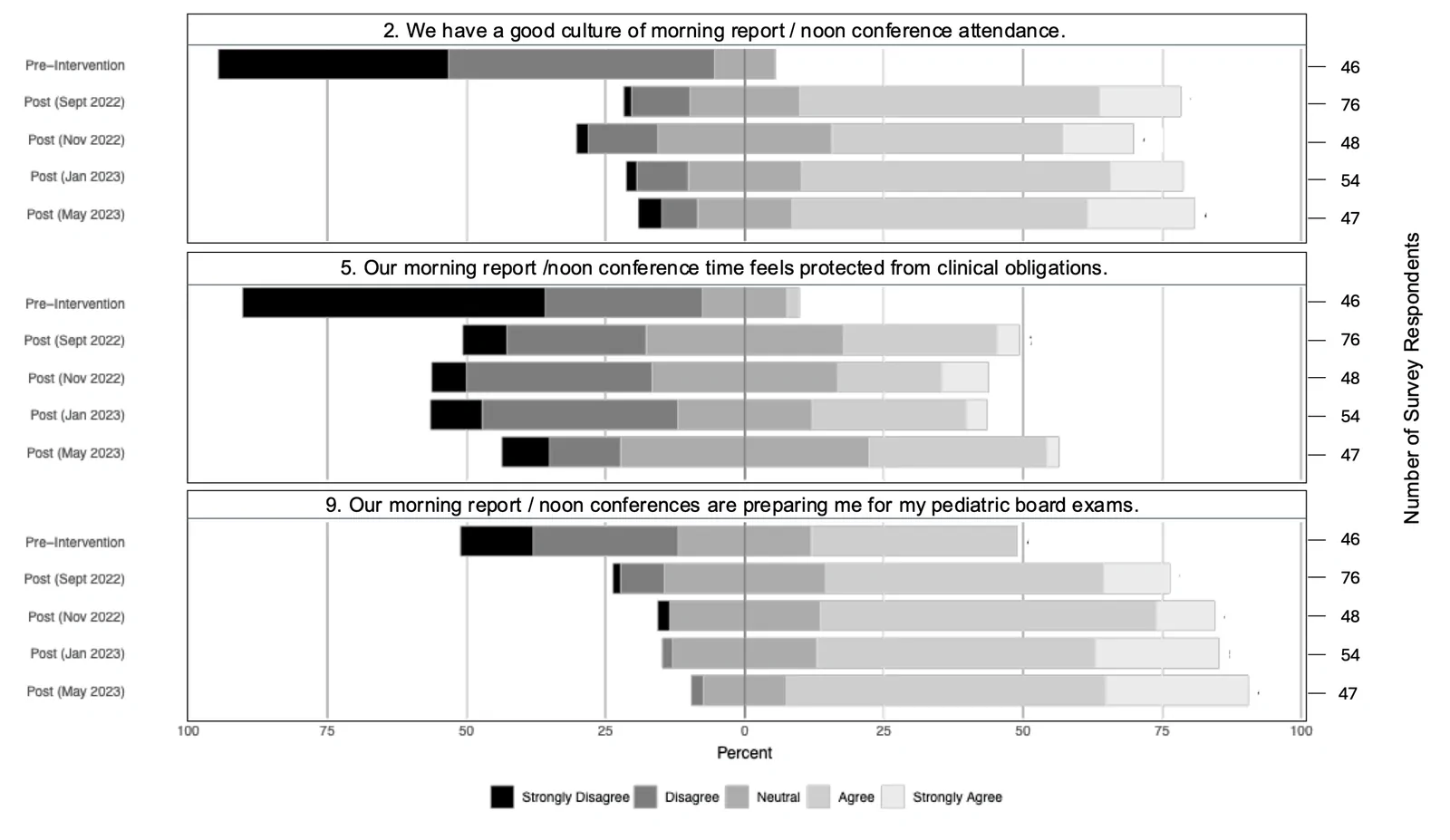
Trends in residents’ level of agreement to three key survey items over time (N=113)

In addition to our measurements, we saw improvements in ACGME Resident Survey scores. Pre-intervention, the annual ACGME survey of our institution highlighted that we were noncompliant in providing “protected time to participate in structured learning activities.” Eight months into our intervention, the number of residents who agreed that our institution provided “protected time to participate in structured learning activities” increased by 19%.

## Discussion

Over the study period, we observed statistically significant and sustained improvements in conference attendance and residents’ perception of conference culture, alongside improvements in ACGME resident survey domains related to protected educational time, which served as an external signal of change. Our goal was to apply KCM to intentionally guide our efforts to improve conference culture within a complex educational system, rather than to isolate the effect of any single intervention.

Outcomes demonstrated a nonlinear pattern, with both attendance and survey scores showing an initial increase followed by stabilization over time. Attendance decreased, but stabilized well above pre-intervention levels, while improvements in perceptions of conference culture were sustained. Viewed through the KCM framework, these patterns are consistent with expected dynamics of organizational change. Early preparatory work emphasizing momentum and urgency, implemented at the start of the academic year, may have contributed to initial engagement as new interns and returning residents were more receptive to new norms. Aligning change efforts with natural transitions can be leveraged to enhance early engagement and adoption of curricular changes [27]. Over time, attendance attenuated, potentially reflecting waning initial enthusiasm, evolving expectations surrounding conference participation, or a diminishing Hawthorne effect, while perceptions of conference culture remained improved. Together, these findings suggest that attendance may serve as a visible and pragmatic process measure of engagement, but an imperfect marker of cultural change.

The return to primarily in-person conferences also likely contributed to early improvements in attendance and engagement. However, in-person format alone does not ensure positive conference culture, as learners may be physically present but disengaged due to preoccupation with their patient census, interruptions from non-urgent messages, or non-interactive material. Resident survey responses highlighted contributors to conference culture, including perceived educational value of conference topics, opportunities for interaction, and faculty presence and role modeling. Importantly, conference culture was influenced not only by those present in the conference room, but also by expectations reinforced outside of it. Support from institutional and interdisciplinary stakeholders was essential to protecting educational time and signaling that conference participation was valued. Pandemic-related disruptions eroded many of the shared expectations surrounding resident conferences. As a result, rebuilding conference culture required more than a shift in format, but deliberate efforts to reset expectations and reinforce norms around education time with those inside and outside the conference room.

As this was a chief resident-led initiative within a time-limited leadership role, KCM provided a framework that balanced structure with adaptability. A strength of KCM is its iterative nature, which resembles a Plan-Do-Study-Act cycle, an evidence-informed quality improvement method that emphasizes continuous reassessment and refinement [28-30]. This flexibility was critical given the inherently dynamic nature of residency training, with residents rotating every two to four weeks and new groups of residents arriving with norms shaped by their prior conference experiences. The KCM framework enabled reinforcement of core expectations during these periods of turnover and during periods of high clinical demand, while also allowing the change leaders to make rolling adjustments in response to ongoing survey feedback. For example, resident input prompted targeted communication with oncoming inpatient senior residents to emphasize their role in modeling and reinforcing conference attendance. This iterative approach likely contributed to the durability of improvements in conference attendance and culture over time.

Our study has important limitations. Survey-related limitations include the development and use of a pragmatic survey tool as opposed to a validated survey instrument, as well as voluntary and anonymous survey participation with variable response rates across surveys, limiting our ability to link individual responses longitudinally and introducing the potential for nonresponse bias. Interpretation of conference attendance is limited by the Hawthorne effect, which we would expect to attenuate over time [31]. Additionally, although improvements in the ACGME survey domains coincided with our interventions, they may have been influenced by concurrent institutional initiatives, as residency training environments are complex and dynamic. Design-related limitations include the single-institution, pre-post design without a control group, which limits generalizability and our ability to attribute observed improvements to our interventions or definitively distinguish the contribution of KCM. Our study also did not include balancing measures, which future work should incorporate to better understand unintended consequences.

Though specific interventions may not be translatable to all institutions, the underlying change management principles of KCM are broadly applicable. Because resident didactics and the clinical environment in which they occur vary widely, educators should begin by engaging trainees to identify locally relevant driving and restraining factors before implementing interventions. Programs should also deliberately map their stakeholder landscape, which often extends beyond the educational office to include hospital and department leaders, nurses, and case managers whose support is critical in protecting educational time and sustaining improvements in conference attendance and culture. Finally, those leading change should visibly model the behavior they seek to promote, reinforcing expectations through consistent participation and presence rather than messaging alone. 

## Conclusions

We observed improvement across multiple indicators of resident educational engagement: conference attendance, residents’ perception of conference culture, and externally reported ACGME survey domains related to protected educational time. These outcomes suggest meaningful improvement in educational culture following post-pandemic disruption. Although our study cannot definitively attribute these improvements to KCM, this study illustrates how a structured change management framework can be applied to help training programs intentionally address conference engagement and educational culture within their own contexts. Further controlled and multicenter studies with longer-term follow-up are needed to evaluate the effectiveness, generalizability, and sustainability of this theory-informed approach.

## Figures and Tables

**Figure 5:**
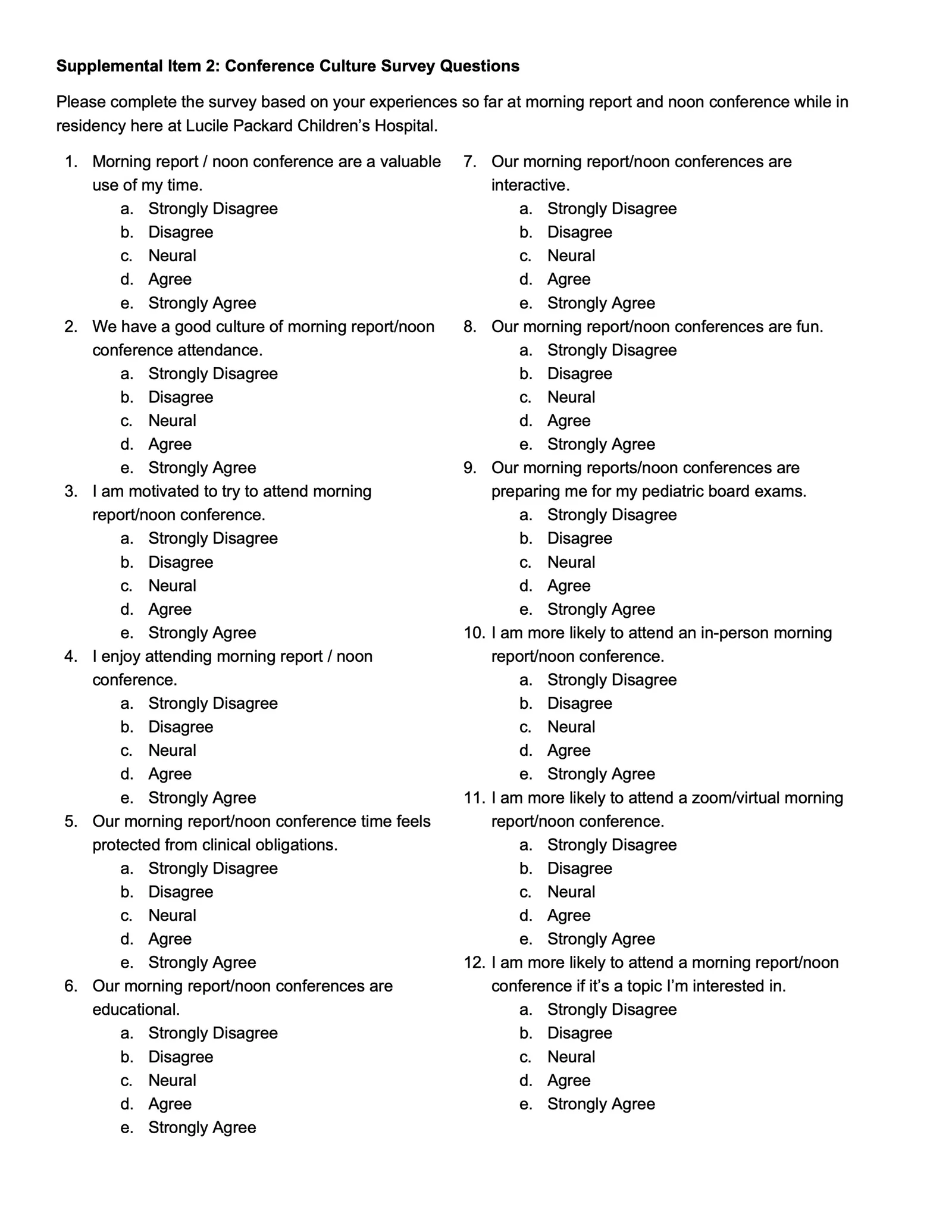
Conference Culture Survey Questions Page 1 of 2

**Figure 6:**
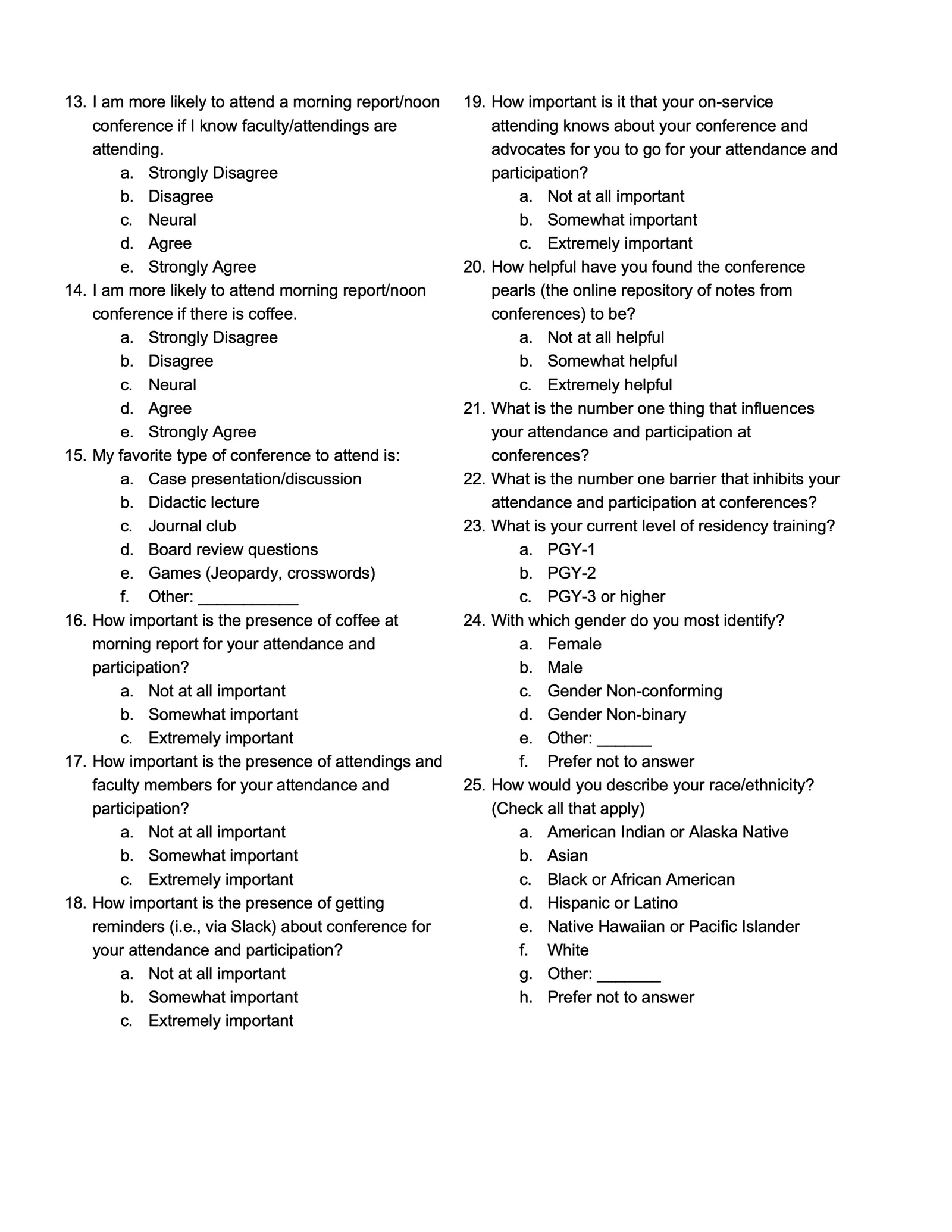
Conference Culture Survey Questions Page 2 of 2

## Appendices

Appendix A 

**Table 3:** Complete list of interventions performed by category and date

Category	Intervention	Date
Conference Content	- Recommended all conferences be given in a case-driven format	Jun ‘22
	- Incorporated board review questions	Jun ‘22
	- Developed an 18-month conference curriculum and invited respective speakers to target high-yield and lower-scoring topics based on an analysis of board exam content and past ITE scores	Oct ‘22
	- Sent each presenter a list of recommended topics for teaching as well as tools and suggestions for making conferences interactive	Oct ‘22
	- Shared standardized e-mail and PowerPoint templates with all conference presenters	Jan ‘23
People	Residents:	
	- Set expectations of conference attendance with new interns at intern orientation and current residents at Step Up trainings	Apr – Jun ’22
	- Rounded up residents in hospital by walking around floors prior to conference start	Jun ’22
	- E-mailed oncoming inpatient senior residents about their role in conference attendance	Dec ‘22
	Attendings:	
	- Identified Attending Physician Champions to advocate among their colleagues	Jun ’22
	- Offered CME to faculty attendees	Sep ‘22
	- Presented initiative to rotation directors (May – Jun ’22), Division Chiefs (Sep ’22), and Department Chair (Jul ’22)	May – Sep ‘22
Environment	- Returned to primarily in-person conferences with hybrid option available for off-site residents	Jun ‘22
	- Provided free coffee at morning report	Jun ‘22
	- Collaborated with stakeholders to pause noisy construction during conference times	Dec ‘22
Clinical Responsibility	- Sent daily Voalte texts to acute care nurses and case management to hold non-urgent questions until after conference time	Jun ‘22
	- Expanded nursing Voalte text reminders to include critical care staff	Oct ’22
Technology	- Taught residents how to send a “quick reply” text on Voalte platform to acknowledge non-urgent message that they would address after protected educational time	Jun ‘22
	- Sent daily reminders to all residents 15 minutes prior to conference start time via Slack	Jun ‘22
	- Sent daily Voalte text reminders to residents and attendings on inpatient service	Nov ‘22

Appendix B
